# The Curious Concept That Almost Nobody Seemed to Care About at First: Virtual Particles in the Post‐War Period**

**DOI:** 10.1002/bewi.202400023

**Published:** 2025-04-15

**Authors:** Jean‐Philippe Martinez

**Affiliations:** ^1^ Max Planck Institute for Gravitational Physics (Albert Einstein Institute) Potsdam Germany; ^2^ Institute of History and Philosophy of Science, Technology, and Literature, Technical University Berlin Berlin Germany; ^3^ Institute for Theoretical Particle Physics and Cosmology, RWTH Aachen University Aachen Germany

**Keywords:** Virtual particles, Feynman diagrams, interpretative debates, quantum field theory, quantum electrodynamics, conservation of energy, history of concepts

## Abstract

Short‐lived, unobservable, and not subject to the usual rules of conservation of energy and momentum, virtual particles—an integral part of the conceptual framework of quantum field theory (QFT)—exhibit a number of curious characteristics which, in recent decades, have in part fueled important discussions about their ontological status. Central to these debates is Richard Feynman's diagrammatic technique for QFT calculations, which provided in the late 1940s the first systematized and generalized description of the concept of virtual particles. At the time, however, the curious characteristics and the ontology of the latter were the subject of little, if any, debate. This article explores how the concept of virtual particles gradually became subject to interpretative scrutiny in the post‐war period. It examines the weight of various aspects of pre‐Feynman developments which once guaranteed a firmer phenomenological anchoring of the scientific practices associated with the virtual particle concept. Subsequently, it shows how the questioning of this concept did not result from a simple assessment of its curious characteristics but was part of a wider critique of the new quantum electrodynamics and Feynman's methods.

## 1 Introduction

“OK, WTF Are ‘Virtual Particles’ and Do They Actually Exist?” In May 2020, the science journalist Morchedai Rorvig posed this question as the attention‐grabbing title of an article in the Canadian‐American magazine *Vice*.^1^ The fact that a topic of this kind was broached in such a popular media underscores, in fact, the growing public interest in a concept that gained prominence following the discovery of the Higgs boson at CERN in 2012 and the subsequent confirmation of the Standard Model. Virtual particles are indeed fundamental to quantum field theory (QFT), the theoretical framework that underpins what is currently our best theory for describing elementary particles and their interactions. The rather amusing formulation of Rorvig's title then reflects two major features of considerations about virtual particles: firstly, in many instances, they turn out to be a rather surprising concept; secondly, they are the subject of debate as to their reality.

**Figure 1:**
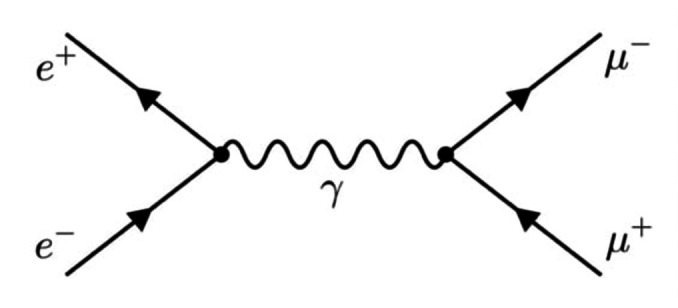
First‐order Feynman diagram for the process $e^{+}e^{-}\to \mu^{+}\mu^{-}$ . An electron‐positron pair is annihilated into a muon‐antimuon pair via a virtual photon γ. Produced with FeynGame. (Harlander et al. 2020.)

According to an image strongly associated with Feynman's diagrammatic method for calculating observables in QFT, the intermediate stages of particle interactions are commonly depicted using virtual particles (see, e. g., Figure 1). Within the framework of this theoretical scheme, Brigitte Falkenburg thus highlithed their highly mathematical nature as well as their non‐independent character—i. e., they are intrinsically tied to interactions.^2^ The author of *Particle Metaphysics*, which offers a systematic analysis of the different concepts associated with the notion of particle, also emphasized that virtual particles are unobservable and cannot be captured by a particle detector and that, contrary to one of the historically most fundamental laws of physics, they are in principle “allowed to violate energy conservation.”^3^ All in all, and even more so as they continue to share basic characteristics with standard particles—such as the properties of mass, energy, spin and charge—virtual particles emerge as a rather curious physical concept.

Hence, as exemplified by Rorvig, discussions surrounding the rather peculiar nature of virtual particles have frequently fueled debates on their realism in recent decades. In this context, one tradition tends to reduce them to mere mathematical fictions, while another defends for their causal and effective nature. Although the breadth and depth of these debates, touching upon far‐reaching physical and philosophical matters, exceed the scope of the present work, it is worth noting from a historical standpoint that they only gained momentum from the 1970s onwards, and mainly within the philosophy of science community.^4^ In particular, Mario Bunge presented in 1970 the first explicit and argued refutation of the reality of virtual particles, basing his reasoning on their behavior in relation to the conservation of energy (to be discussed further in section 3). His approach, which advocated the abandonment of the concept, then stands as a remarkable illustration of a surprising—at least at first sight—shift.^5^ Indeed, the most common representation of virtual particles, which already displayed all their intriguing characteristics, had long been established by Feynman's work in the late 1940s. But during this interval, while opinions on the realism of the phenomena studied and their representations may have been expressed in the course of various physical and philosophical investigations, the specific nature of virtual particles was never really the subject of any significant concern.

The aim of this article, therefore, is precisely to discuss the mechanisms that first prevented, then encouraged, virtual particles from becoming an object of increasingly important interpretative concern. It first traces the pre‐Feynman history of the concept, to show how it ensured by the late 1940s a firm phenomenological anchoring of the scientific practices associated with its use. Secondly, it examines how a growing interest in the question of the validity of QFT gradually led to the spotlight being turned on the interpretation of virtual particles themselves.

## 2 Towards Feynman's Virtual Particles: Theoretical and Phenomenological Grounding

### 2.1 Early Developments: Virtual Transitions to Intermediate States

The pre‐Feynman history of the virtual particle concept, particularly in the early stages of quantum electrodynamics (QED)—the specific quantum field theory that describes photon‐mediated interactions—has recently been studied in depth by Markus Ehberger.^6^ He has notably emphasized the major role played in the establishment of their physical foundation by Paul Dirac's 1927 radiation theory.^7^ The theoretical physicist's perturbative approach to the radiation field led to the appearance of new second‐order terms in the perturbative series, which he understood as transitions from an initial state to a final state via an “intermediate” state for the concerned system.^8^ These transitions were first described as “virtual” by Chandrashekara Raman at a symposium on molecular spectra and molecular structure held in Bristol in September 1929 by the Faraday Society.^9^ He used the term while discussing the effect that now bears his name, the Raman effect, which he had just discovered a year before. Within Dirac's theoretical framework, Raman suggested that “the transition of the molecule assumed for the purpose of the calculation is a purely virtual one which cannot actually occur.”^10^ Adopting a rather critical and anti‐realist stance that emphasizes the mathematical nature of transitions to an intermediate state, his reasoning was anchored on the observation that the energy of the visible light used in the experiments was insufficient to allow the molecule under study to transit through the intermediate state determined by Dirac's theory. And with good reason: Dirac himself had acknowledged transitions through an intermediate state as the result of two processes, “one of which must be an absorption and the other an emission, in neither of which is the total proper energy even approximately conserved.”^11^

These circumstances stimulated early discussions pertaining to realism. For instance, Yakov I. Frenkel responded to criticisms akin to Raman's by arguing about the “definite duration” of intermediate states and demonstrating the harmony of complete processes with the conservation of energy.^12^ Indeed, in contrast to individual absorption or emission events, when considering the actual time evolution of the complete system using the full Hamiltonian—which includes all interactions—energy is conserved. Moreover, as Ehberger has clearly shown, in the early years following Dirac's developments, the terminological choices of many theoretical physicists often reflected their views on related discussions.^13^ Figures such as Ivar Waller and Léon Rosenfeld regarded intermediate states as purely formal entities in the early 1930s, while others, like Dirac and Igor Tamm, tended to grant them a physical character. However, consistent deliberations remained confined to a brief period after the introduction of the concept of virtual transition, and its ontology never really became a subject in its own right.

In fact, Dirac's mathematical modelling and its verbal representation, particularly expressed by the idea of transitions via an intermediate state, quickly established themselves as an invaluable tool for the quantum physics community.^14^ Subsequently, by the mid‐1930s onwards, the prevailing attitude with regard to Dirac's theory simply leaned towards pragmatism: “the interpretation of the formalism was pushed into the background, while the application of the calculational scheme gathered prominence.”^15^ This trend was especially noticeable within a research group centered around Werner Heisenberg in Leipzig. Two of his doctoral students, Hans Euler and Bernhard Kockel, leveraged Dirac's scheme for the theoretical evaluation of electron‐electron and light‐by‐light scattering processes.^16^ Their efforts played a crucial role in the development of the perturbative scheme in QED, but also in instituting the terminology of “virtual transitions” and “virtual intermediate states,” years after Raman's initial, albeit inconspicuous, usage of the term “virtual.”^17^ Interestingly, it should be noted that this was achieved by highlighting in various cases—although rather indirectly—the feature of energy non‐conservation.^18^ This association would then be further emphasized in the realm of nuclear physics.

### 2.2 Nuclear Forces: Extending the Class of Phenomena

In the late 1930s, the field of nuclear physics actually provided some of the most insightful and straightforward comments on the meaning of virtuality for transitions to intermediate states. They were motivated in particular by the announcement, in 1936, of the discovery of a new particle during experiments on cosmic rays conducted by Carl Anderson and Seth Neddermeyer.^19^ This discovery drew the attention of Western physicists to Hideki Yukawa's postulate, formulated at the end of 1934, of a particle of similar mass.^20^ Yukawa's proposal formed the basis of an innovative theory of nuclear forces, in which this new particle was conceived as being emitted by one nucleon and reabsorbed by another. Interpreted as the mediator of interaction between nucleons, this particle was in many respects the first to be directly postulated as virtual.^21^ Resulting from the application of the perturbative scheme of QED to the study of nuclear forces—marking its initial extension into QFT—it was indeed understood as a transient and unobservable entity which, in the course of its “exchange” between nucleons, temporarily violates the conservation of energy.

This novel development in nuclear physics prompted a few scientists involved in later developments to give a little more thought than usual to the concept of virtual transitions. All the more so as the term “virtual” itself, first introduced into the field by Heisenberg and later adopted by Yukawa, began to be used more consistently in this context.^22^ Comments made by Homi Bhabha and Giancarlo Wick in 1938 are noteworthy. They helped to establish more forcefully the centrality of energy non‐conservation in the definition of virtual transitions to an intermediate state. As asserted by Bhabha:

Of course, the emission or absorption of a U‐particle [Yukawa's] only takes place observably when it is consistent with the conservation of energy. In other cases the emission or absorption is merely virtual, as an intermediate state, as is the case in the quantum theory of radiation.^23^

Wick, who also specified that “emission and absorption processes, which would be contrary to the energy principle […] are called, therefore, virtual transitions,” took the reasoning a step further. He explained “how it comes about that the energy principle is respected,” based on the fact that non‐conservation is confined by Heisenberg's time‐energy uncertainty relation, ΔEΔt≥ℏ/2 , which in turn imposes a short lifetime on virtual processes.^24^ It is worth noting that in the 1930s, Wick's contribution represented the most explicit expression of the connection of virtual processes with the uncertainty relations, helping to build a narrative that would later become one of the most frequently invoked to explain the non‐conservation of energy in virtual transitions, but also by virtual particles.^25^ Nonetheless, in the meantime, such a direct address of the potential questions surrounding the intriguing nature of the latter remained relatively isolated.

Two key categories of factors justify this lack of interest in potential implications. On the one hand, in addition to and contributing to the pragmatic attitude adopted by the users of Dirac's perturbative scheme, virtual processes were in fact increasingly legitimized, from the mid‐1930s onwards, by major explanatory successes. In parallel with successes in QED, Yukawa's theory was pivotal in providing the sole coherent explanation of the strong nuclear force, which binds the nucleons inside the nucleus. On the other hand, this dynamic was accompanied by an increasing growth in the phenomenological grounding of the concept of virtual particles. Intriguingly, this was largely based on a confusion that persisted for a decade and significantly shaped the ensuing research into Yukawa's theory of nuclear forces. In fact, the Japanese theorist had not postulated the particle observed by Anderson and Neddermeyer in 1936, known today as the muon, but a different one, the pion. Be that as it may, for around a decade, the majority of the community believed them to be the same and referred to them as “mesons.” This belief, apart from aspects related to their comparable masses, was also based on Yukawa's clear stipulation in his original paper that the meson he had postulated might have “some bearing on the shower produced by cosmic rays.”^26^

The effective discovery of the muon in 1936 and its association with Yukawa's pion then established a clear link between the latter and the observable world: if given sufficient energy, a “virtual emission” could become a “real emission” and therefore be detected, to use a terminology used by Walter Heitler, for instance.^27^ This situation conferred upon the scientific community an impression of the empirical adequacy of Yukawa's theoretical model, which is reflected in the fact that meson physics has rapidly become a central element in cosmic ray studies to account for various phenomena.^28^ Initially artificial and therefore plagued by various theoretical and experimental inconsistencies,^29^ this impression was eventually upheld, when in the early post‐war period a new theoretical model opting for a two‐meson solution was developed—it conceived of the muon has the decay product of the pion—and experimental confirmation of the existence of the pion was finally established in 1947 by Cecil Powell, César Lattes, and Giuseppe Occhialini.^30^

The context of meson theory also witnessed the qualifier “virtual” being more directly applied to the particles themselves, beyond just transition processes.^31^ Then, while their originality was recognized, they were not generally perceived as problematic, or at least not called into question. As a tool, at a minimum, they had proved their worth, and interpretative discussions, including ontological ones, did not gain traction.^32^ And in fact, if the adjectives “real” and “actual” started to be used in contrasts to “virtual,” this could only serve to denote something that was precisely what its name implied.^33^ On the whole, the pragmatic approach that physicists had quickly adopted towards the concept in formation of virtual particles and related processes was simply validated by their increasing explanatory and phenomenological power.

### 2.3 Feynman Diagrams: Reconceptualization, Generalization, and Systematization

The period of the Second World War, while causing a significant pause in the progress of QED and meson theory, was followed by a flurry of groundbreaking developments that completely transformed our approaches to the various problems tackled so far in relativistic quantum field theory. In addition to the emergence of the two‐meson theory, Shin'ichiro Tomonaga, Julian Schwinger, and Richard Feynman made significant contributions by developing manifestly covariant formulations of QED in the late 1940s. Remarkably, their work helped establish the so‐called renormalization techniques, which found their first rigorous and coherent formulation in the work of Freeman Dyson and played a crucial role in solving an enduring issue in QED: the problem of infinities.^34^ It was in this postwar context that Feynman introduced his innovative method for calculating observables, now known as Feynman diagrams.^35^ These diagrams provide a pictorial representation of scattering processes: external lines denote the initial and final states, while internal lines, which correspond to intermediate states, have been firmly associated with virtual particles according to Feynman's original terminology (see Figure 1).^36^ Through consistently applied Feynman rules, these diagrams can be easily converted into mathematical expressions that yield the scattering amplitudes for the process under study. In fact, these scattering amplitudes, which predict the probability of different outcomes in particle collisions, are obtained according to the path integral formulation by considering the sum of the contributions from all possible diagrams describing a particular scattering reaction.^37^

The reconceptualization of virtual particles this new approach thus introduced—representing them in individual diagrams while emphasizing their mathematical meaning as a collective only—was all the more profound as their behavior in relation to conservation laws was reconsidered. The relativistic expression relating total energy with rest mass and momentum, $\sqrt{E^{2}-{\vec{p}}^{2}c^{2}}=mc^{2}$ , plays a central role in this scheme. Observable particles, accounting for the initial and final states, are deemed to be “on‐shell,” meaning they strictly comply with this expression. On the other hand, virtual particles, known as “off‐shell,” are not required to obey the energy‐momentum relation, which in turn allows the exact conservation of energy and momentum at each vertex of a Feynman diagram. Feynman's approach then proved successful and gradually imposed itself as the standard calculation technique for high‐energy phenomena throughout the second half of the twentieth century.^38^ For the first time, it went on to provide the scientific community with a visual, generalized, and systematized description of the concept of virtual particles, with Feynman diagrams being applicable, at least in principle, to all types of relativistic quantum fields.

A historian or philosopher might expect this new formalization of the concept of virtual particles, as well as its prominent role in the new QED, to have immediately prompted specific conceptual analyses and discussions of its curious characteristics. However, based on our research findings, it appears that virtual particles, in and of themselves, have not received much attention. Instead, much of the interpretative discussion of Feynman's method has initially taken place in the context of what David Kaiser termed “the Feynman‐Dyson split”: “[the] tension between Feynman's and Dyson's positions, with their varying emphases on ‘intuition’ versus derivation, physical pictures versus topological indicators.”^39^ Hence, the main question revolved around the ontological status attributed to Feynman diagrams. Feynman initially considered them to be direct representations of real phenomena, whereas Dyson regarded them as solely having instrumental value. Then, even though this conflict echoes early considerations about transitions to an intermediate state, it did not extend to virtual particles proper, which remained largely untouched by in‐depth ontological examination. Indeed, the disagreement between Feynman and Dyson had much less to do with the possibility of deconstructing Feynman diagrams into their elementary constituents than with their overall ability to represent phenomena that actually occur. This is all the more true given that, despite the aforementioned process of reconceptualization, generalization, and systematization, the concept of virtual particles was not yet intrinsically linked to Feynman's approach. After all, the original idea behind virtual processes—namely the transition to an intermediate state—had played a key role in the foundations of the new diagrammatic approach to QED. As a result, the concept of virtual particles was seen as having a well‐established independent pre‐history that continued to lend these theoretical entities solid explanatory and phenomenological power. Given this background, the introduction of Feynman diagrams, which primarily unveiled themselves as a mathematical tool, was not a compelling reason to alter perceptions of virtual particles.

## 3 Making Virtual Particles an Object of Interpretative Concern

### 3.1 Virtual Particles Do Not Respect Energy Conservation: So, What?

In fact, it was not until the mid‐1950s that the concept of virtual particles began to receive attention. Following the success of the new QED, it emerged the need to assess it, as evidenced by a series of lectures held at the University of Birmingham on 13 and 14 December 1954, featuring Rudolf E. Peierls, Abdus Salam, Paul T. Matthews, and Gordon Feldman.^40^ Published in 1955, this “survey of field theory” included an introductory lesson prepared by Peierls. Remarkably, this section contains relatively detailed comments, unusual for the content published at the time, on the notion of virtuality in quantum physics. Peierls began by stating that “[i]t is a general feature of quantum mechanics that the effect of small coupling terms in the second and higher order is described in terms of successive ‘virtual’ transitions through intermediate states of the system,” before adding that “[t]he term ‘virtual’ here serves has a reminder that we have to consider intermediate states in which energy is not conserved.”^41^ In the explanation he then gave of the unconventional behavior of virtual processes in terms of energy conservation, Peierls definitely echoed positions already held in the 1930s. For him, the Heisenberg uncertainty relations delimited the short lifetimes of intermediate states and their ability to circumvent conservation laws.

Peierls’ adherence to a pre‐Feynman “old‐school” perspective—after all he was born in 1907 and became a major actor of the developments of quantum theory in the 1930s—is particularly noteworthy, especially considering that he was the first to explicitly link in his reasoning the transitions to an intermediate state with the behavior of particles during quantum tunneling. Indeed, this association adds a unique dimension to the debates, since the notion of virtuality was independently introduced for the second case by Guido Beck in the early 1930s, in a context strictly distinct from that of perturbative calculations.^42^ Then, overall, this situation led Peierls to adopt a metaphor, originally articulated by Frenkel in 1934 and later taken up by Georges Gamow and Charles L. Critchfield in 1949, to address the issue of energy conservation: “Crudely speaking, a system may borrow an amount of energy for a short time, provided the loan is returned before it is possible to discover the amount is missing.”^43^ This image, which indirectly confers a certain ontological weight on virtual particles, also undermined the problem of the conservation of energy, by treating it as a simple and convenient internal arrangement. Following the publication of Peierls’ introductory lesson in *Reports on Progress in Physics*, a fairly influential journal that ensured wide circulation and large readership, the “borrowing” metaphor gained in popularity. It found its way into discussions well into the second half of the twentieth century, even in contexts more directly related to Feynman diagrams.^44^

Whatever its overall impact, this contribution stands out above all for its influence on the reflections of the first philosopher of science to take a direct interest in the concept of virtual particles. Mary Hesse, in her seminal book *Forces and Fields*, published in 1961, dedicated two pages to this very subject. In doing so, she directly referenced Peierls, thereby acknowledging that the term “‘[v]irtual’ has always to be understood as non‐energy‐conserving, and therefore short‐time.”^45^ Yet, Hesse's philosophical analysis transcended this mere observation, resonating closely with Falkenburg's recent assessment presented in the introduction. With regard to the characteristic of unobservability, she notably recognized that the dichotomy between “virtual” and “real” emissions of radiation is “in conformity with the quantum mechanical convention that ‘reality’ is ascribed only to what is detectable in a classical sense, that is, to radiation which can produce observable effects on photographic plates, and so on.”^46^ Furthermore, Hesse elucidated the distinction between real and virtual as an expression of the division between “free” particles and “bound” particles, echoing Falkenburg's observation that virtual particles are tied to interactions.

However, it is somewhat surprising to observe Hesse's cautious stance on profound interpretative matters. When considering the potential ramifications of the question of energy conservation on realism, she simply suggested that “it does not seem necessary to insist on this.”^47^ Her rationale stemmed from the uncertainty principle, which effectively precludes us from scrutinizing in detail any breach of such a law.^48^ In Falkenburg's terminology, virtual particles are simply “allowed” to violate energy conservation; nevertheless, as Hesse seemed to suggest, this does not necessarily imply that they actively contravene it.^49^ Moreover, while the British philosopher acknowledged that the description of QFT in terms of “virtual interactions” might be a mere “pictorial representation of the first‐order terms of the expression for interactions of the source particles,” she did not cast doubt on the reliability of of this approach.^50^ In fact, she even suggested that, due to the divergencies found in the formalism, this approximation could “turn out more reliable than the whole expansion.”^51^

On the whole, despite being the first philosopher to address various interpretative issues related to virtual particles, Hesse ultimately embraced the pragmatic approach of physicists, which remained the prevailing norm in the early 1960s. This attitude was strongly reflected in the belief that the “potentialities of the general field theory naturally lead to hope that the gravitational field can be shown to be a particular case of interaction by means of virtual particles.”^52^ Theoretical physicist Kenneth W. Ford eloquently expressed the powerful extension of Hesse's sentiments in 1963 in his book *The World of Elementary Particles*. To him, the idea of the virtual particle provided “a very beautiful example of the workings of the Heisenberg uncertainty principle at the elementary level, and […] a clue, not only to the nature of the strong interactions, but to the nature of *all* forces and interactions.”^53^ Ford's conviction in this regard was significantly bolstered by the success of Yukawa's theory, “enough to make us believe” in the image of protons surrounded by a field of virtual pions.^54^

As these examples illustrate, the concept of virtual particles soon received increasing attention as the particle physics community expanded throughout the 1950s, featuring prominently in reviews and textbooks. Yet, as can also be seen in the above cases, Feynman diagrams and the reconceptualization of the virtual particle concept they introduced were not at the center of these discussions. However, the latter did possess a general character—through the emphasis placed on the meaning of the word “virtual,” or the broad physical implications expressed by Hesse and Ford—which may have contributed to the perception of a completely unified concept across different theories. Even Ford, when introducing Feynman diagrams in his book, reffered to virtual particles as those originally introduced in the context of Yukawa's theory.^55^ Overall, while the intriguing characteristics of virtual particles kept on being duly recognized in the litterature, they firmly retained their strong explanatory and phenomenological power, while possible philosophical ramifications did not arouse significant concern. This state of affairs would gradually change over the following decades, as Feynman diagrams came under increasing scrutiny.

### 3.2 The Anti‐QFT Turn

One of the most amusing manifestations of the influence of Peierls’ 1955 insights on virtuality can be found in a letter addressing the theme of extrasensory perception sent in 1979 to *Science* by physicist Henry Margenau and psychologist Lawrence LeShan.^56^ Here, a convergence of ideas regarding intermediate states and quantum tunneling was invoked to propose that “physics itself tolerates curious expectations, or at any rate, it considers phenomena which alter the usual conception” of the basic principle of energy conservation. Beyond the uncommon juxtaposition of physical concepts, the plausibility of Peierls’ significant influence lies in the discussion of these concepts specifically in relation to energy conservation. This was indeed an unusual stance for Margenau, whose contributions to quantum theory typically underscored the notion of unobservability to justify virtuality. This is exemplified in two philosophically oriented publications from 1954, focusing on causality, where he asserted that since “[t]he occurrence of pair production and annihilation remains hidden, […] the whole process is called ‘virtual pair production.’”^57^ It is then worth noting that these papers constituted one of the earliest philosophical assessments of Feynman's approach to QED, for which Margenau positively advocated strict consistency with the principle of causality.

Margenau's adherence to the notion of unobservability in the definition of virtual particles resurfaced in the 1970s, when he was invited to contribute an entry on “fields” to the *Encyclopædia Britannica*. In the segment dedicated to relativistic quantum fields within the original manuscript, he explained: “It can happen that a photon will be emitted and absorbed before it can be detected. Such a photon is termed a virtual photon, as opposed to a physical photon which can in principle be detected.”^58^ However, the tenor of his subsequent elaborations sharply contrasted with his favorable assessment of Feynman's work in the 1950s. Indeed, the introduction of the concept of virtual particles and its direct connection with Feynman diagrams—as “[e]ach Feynman diagram is a pictorial representation of the manner in which virtual particles can be emitted and absorbed”—was followed by a lengthy exposition of the various limitations of the mathematical formalism of QFT, along with explicit advocacy for the pursuit of alternative theories.

Margenau's newfound cautious stance towards QFT mirrored a broader trend that had previously emerged within particle physics. Despite the many successes achieved since the late 1940s, part of the scientific community had gradually began to harbor doubts regarding the validity and applicability of renormalization techniques.^59^ These doubts were notably motivated by the non‐renormalizable character—it predicted infinities at higher order that could not be removed—of the Fermi theory of β‐decay, which was the standard model for weak interactions at the time.^60^ Additionally, general skepticism among physicists was fueled by the early recognition that the perturbation theory is effective only in describing small interactions.^61^ Against this backdrop, the prospect of alternative approaches gained momentum. One noteworthy example is S‐matrix theory. Initially developed in the 1940s by Werner Heisenberg to tackle divergences in QFT, it was overshadowed by renormalization techniques before undergoing a significant resurgence from the late 1950s, driven by the growing urgency of questions related to strong interactions.^62^ Ultimately, concrete advances in understanding hadronic matter have also contributed to underscore the imperative to address the limitations of QFT. It became particularly pressing starting in the mid‐1960s, especially with the recognition that nucleons are not fundamental particles and the formulation of the quark model by Murray Gell‐Mann and George Zweig, independently.^63^

As the foundations of QFT began to face scrutiny, exposing their weaknesses, Hesse's neutrality towards interpretative issues concerning virtual particles gradually became untenable. In this overarching trajectory, it is not surprising to encounter figures such as Margenau and, as will be explored further, Bunge. Indeed, as trained physicists with a genuine penchant for philosophical inquiry, they were, in their own way, among those “quantum dissidents”—if not “hippies” in the case of the former, due to his interest in extra sensorial perception—who helped move the field of quantum foundations from the margins of physics into its mainstream in the post‐war era, and usher in the age of quantum information.^64^ They had a deep understanding of interpretive issues, and the challenges posed by particle physics gave them a new opportunity to exercise their critical faculties. In the case of Margenau—who, in 1970, was one of the founding editors of the journal *Foundations of Physics*—this led to an unconventional interpretation of the role of virtual particles within the framework of Feynman diagrams. As previously mentioned, in his contribution to the *Encyclopædia Britannica*, the diagrams were not merely portrayed as representations of standard particles scattering processes, but as depictions “of the manner in which virtual particles can be emitted and absorbed.”^65^ This articulation of a specific, intertwined relationship between the concept of virtual particles and Feynman's diagrammatic approach signified a change of perspective, departing from the view that had hitherto attributed to the former a solid phenomenological grounding independent of the development of the latter.

In fact, a significant step in this direction had already been taken in 1970 by Bunge, when he had described the genesis of the concept of virtual particles as “the attempt to assign a physical meaning to every term in a perturbative expansion, or, equivalently, to give a literal interpretation of every Feynman diagram.”^66^ However, unlike Margenau, Bunge did not hesitate to confront the potential implications of the concept taken in isolation. In this direction, he focused precisely on non‐conservation of energy—a feature that, as we have observed, did not have as much primacy as unobservability for Margenau—which he invoked as central to the evaluation of QFT. Articulated in a concise two‐page paper, Bunge's relatively simple argument can be encapsulated as follows: since virtual particles do not adhere to energy conservation, they cannot be deemed real; as they are mere fictions, they have no place in physical theory, since ultimately rendering our field theories non‐physical. The curious characteristics of virtual particles—a “weird metaphysical notion” in Bunge's words—previously overlooked amidst successes justifying a purely pragmatic approach, were suddenly thrust into the spotlight as grounds for abandoning the concept.^67^ Virtual particles had at last become an object of philosophical concern, all the more so as they allowed a pointed critique of quantum field theories, which Bunge accused of “involving false or meaningless premises” and consisting “in hanging one fiction from another in the style of Ptolemaic astronomy.”^68^

## 4 Concluding Comments

At first, Bunge's radical call for the abandonment of virtual processes and particles failed to meet with widespread resonance. In part, this may be due to the development of the Weinberg‐Salam model in the late 1960s, which proposed a unified and renormalizable model of electroweak interactions, followed by the advent of quantum chromodynamics (QCD) in the 1970s, providing a coherent framework for understanding hadronic interactions.^69^ These advances gradually made criticism of QFT less pressing for particle physicsists by addressing many of the key‐challenges they faced. This new situation even pushed the S‐matrix approach into the background and streamlined the completion of the Standard Model. Nonetheless, although the Weinberg‐Salam model and QCD resolved many immediate concerns, they did not eliminate the intrinsic weaknesses of QFT that had been highlighted until then.

The skepticism initially expressed by Margenau and Bunge then gained specific momentum among philosophers of science, when in the late 1970s and early 1980s they seized upon tensions within the particle physics community as fertile ground for discussing Thomas Kuhn's thesis on scientific revolutions.^70^ In 1977, Kristin Shrader‐Frechette analyzed the prevailing paradigm in high‐energy physics, suggesting that the field was approaching a state of “crisis.”^71^ Her reasoning was based on the identification of various conceptual problems related with the notion of elementary particles, which, in line with our discussion, “ha[d] not been spelled out in any great *philosophical details*.”^72^ R. Edward Hendrick and Anthony Murphy's response to this paper, which warned against the “illusion of crisis,” then prompted a rebuttal from Robert Weingard, who focused in particular on their discussions regarding the concept of virtual particles.^73^ Weingard presented what remain the most compelling arguments for and against the reality of virtual particles. The former revolves around the interpretation of annihilation and creation operators in QFT, suggesting a shared ontology for real and virtual particles. The later, often referred to as the “superposition argument,” relies on the fact that the perturbative expansion, leading to a superposition of contributions from different Feynman diagrams, does not permit a sharp definition of the number or type of virtual particles.

Weingard's 1982 contribution, which paved the way for many subsequent debates in the philosophy of physics, in fact reflects a complete break with past considerations that were once essential to understanding the physics community's attitude towards virtual particles. On the one hand, the issue of energy conservation, which had previously played a central role in defining the concept of virtual particles, had faded into the background and now occupies a less important place in contemporary discussions.^74^ Perhaps with good reason, since Hesse had already demonstrated its potential irrelevance in 1961. On the other hand, in line with the insights of Bunge and Margenau in the early 1970s, the focus had decisively shifted towards reducing virtual particles to their role as internal lines in Feynman diagrams. This development definitively overshadowed the time when the scientific community placed confidence in the concept of virtual particles as intermediate states, recognizing its explanatory and phenomenological power. A confidence that had actually contributed to enabling Feynman in the late 1940s to develop one of the most innovative and ingenious calculational techniques of the twentieth century, marking in a way one of the greatest triumphs of pragmatism in the history of modern physics.

Ehberger, drawing precisely on his experience with virtual particles, recently argued “for conceiving of concepts as tools embedded in historically situated constellations and practices, which in turn means that their application, fruitfulness, and epistemic power depends on the framework in which they are put to use.”^75^ The present study is not only an illustration of the malleable nature over time of this dynamic interplay, but also resonates with the call for historians to shift “attention from the characteristics of virtual entities to their functions and the way in which their representations are put to use.”^76^ Indeed, while the enigmatic nature of virtual particles may understandably attract attention and prompt questions about their reality, history also demonstrates that physicists can easily accommodate such a situation. All the more so as, in the end, even the scrutiny of the validity of the concept of virtual particles—by way of a deep integration into a critique of Feynman diagram techniques led mainly by philosophers of science—appears more as a symptom of a crisis of confidence in our theories rather than a genuine concern with a seemingly curious concept.

## Footnotes

n1 Rorvig 2020.

n2 Falkenburg 2007, on 236.

n3 Ibid.

n4 Readers interested in these debates are invited to consult some of the most recent contributions: Fox 2008; Valente 2011; Jaeger 2019. For a full review, see also: Ehberger 2024a (section 2.2).

n5 Bunge 1970.

n6 Ehberger 2020; Ehberger 2022; Ehberger 2024a.

n7 Dirac 1927a and b.

n8 It is worth noting that, as Ehberger has also developed, the notion of intermediate state existed before Dirac, notably with the development of the Kramers‐Heisenberg dispersion formula in 1925. Kramers and Heisenberg 1925; Ehberger 2024 (sections 4.3 and 4.4). However, it was Dirac who first provided a sound theoretical basis for transitions to an intermediate state within the framework of quantum mechanics.

n9 Raman's specific reasons for employing the term “virtual” remain somewhat obscure. However, it is worth noting that the term was gaining traction in quantum physics at the time. This was due to Niels Bohr, Hendrik Kramers, and John Slater introducing concepts like “virtual oscillators” and “virtual radiation field” in 1924, as part of their innovative yet unsuccessful approach to radiation. This theory, known as BKS theory, is discussed by Sofia Guilhem Basilio in this issue of *Berichte zur Wissenschaftsgeschichte* particularly regarding its reception by Erwin Schrödinger. For further exploration of the introduction of virtuality in this context and its potential implications for future developments, see: Ehberger 2024a (part I); Martinez 2024; Blum and Jähnert 2024.

n10 Raman 1929, on 790.

n11 Dirac 1927b, on 712.

n12 Frenkel 1929a, on 758; Frenkel 1929b.

n13 Ehberger 2022; 2024a (part II).

n14 Ehberger thoroughly examined what he called the “picture of subsequent transitions,” i. e., Paul Dirac's “verbally explicit description of the light–matter interaction in terms of subsequent and temporally ordered virtual (modern terminology) transitions,” and its influence on developments in QED. Ehberger 2020, on 564.

n15 Ibid., on 267.

n16 Euler and Kockel 1935; Euler 1936.

n17 Unlike Raman, the Leipzig group's use of the term “virtual” did not carry a negative connotation. For initial insights into the meaning of virtuality in these various contexts, see: Martinez 2024.

n18 For example, Euler wrote: “These partial processes to the *‘virtual’* intermediate states do not require energy conservation like the overall process (I→ n) to the *real* final state [Diese Teilprozesse zu den *‘virtuellen’* Zwischenzustände verlangen keine Energieerhaltung wie der Gesamtprozeß (I→ n) zum *wirklichen* Endzustand].” Euler 1936, on 415. The emphasis here was placed by Euler himself, stressing that the conservation of energy comes as a feature of virtuality.

n19 Anderson and Neddermeyer 1936.

n20 Yukawa 1935. On Yukawa's theory, and its impact on 1930s physics, see, e. g., Brown 1981.

n21 Note however that Yukawa initially described this particle as a “heavy particle” and did not use the terminology of the “virtual.”

n22 Heisenberg [1935] 1984; Yukawa and Sakata 1937.

n23 Bhabha 1938, on 117.

n24 Wick 1938, on 994.

n25 Ehberger carefully investigated the early occurences of the link between virtual processes and uncertainty relations. He has shown that, prior to Wick's significant contribution, besides Frenkel's above‐mentioned 1929 paper, this link had notably been sketched by Lev Landau and Rudolf Peierls’ in their 1931 renowned article on quantum field measurement. Frenkel 1929b; Landau and Peierls 1931; Ehberger 2024a (section 10.2.3). For modern occurences of the idea that the energy non‐conversation of virtual particles is framed by Heisenberg's time‐energy relation, see, e. g.: Halzen and Martin 1984, on 7; Aitchison and Hey 2013, on 17–19 and 172.

n26 Yukawa 1935, on 57.

n27 Heitler 1938, on 382.

n28 On the interactions between cosmic ray physics and Yukawa's meson theory, see: Cassidy 1981; Monaldi 2005.

n29 See, in particular, Monaldi 2005.

n30 Marshak and Bethe 1947; Lattes et al. 1947.

n31 For example, one of the earliest occurrences was given by Heisenberg who spoke in 1939 of “virtual Yukawa particles [virtuellen Yukawa‐teilchen]” in the context of cosmic ray phenomena. Heisenberg 1939, on 83.

n32 A notable, but isolated, counter‐example is Rosenfeld who, in his 1948 monograph on nuclear forces, reaffirmed his critical stance from the early 1930s, stating: “If, nevertheless, we wish to use the corpuscular language, we must therefore not lose sight of the symbolic character of the imagined transport of energy by photons; and of this we are reminded by the phrase ‘virtual photon transition.’” Rosenfeld 1948, on 13.

n33 See the argument developed in Martinez 2024.

n34 See, e. g.: Rueger 1992; Schweber 1994.

n35 Feynman 1949.

n36 Ibid.

n37 For technical as well as historical considerations on calculations using Feynman diagrams, see Harlander and Martinez 2024.

n38 On the dissemination of Feynman diagrams, see Kaiser 2005.

n39 Ibid., on 263.

n40 Peierls et al. 1955.

n41 Ibid., on 428.

n42 Beck 1930. More in Martinez 2024.

n43 Frenkel 1934; Gamow and Critchfield 1949; Peierls et al. 1955, on 428. It is not surprising to find Frenkel as the instigator here, as his scientific style often featured the repeated use of metaphors. See Kojevnikov 1999.

n44 Also developed at length by Peierls in his book, *The Laws of Nature*, this metaphor notably found its way—to cite just one of many examples—into the influential particle physics textbook of Francis Halzen and Alan D. Martin, *Quarks and Leptons: An Introductory Course in Modern Particle Physics*. Peierls 1955; Halzen and Martin 1984, on 7.

n45 Hesse 1961, on 278.

n46 Ibid., on 277.

n47 Ibid.

n48 In 1959, Soviet physicist Yuri V. Novozhilov similarly advanced this argument, among other discussions on virtual particles, in his book *Elementarnye Chastitsy* (Elementary Particles). Nevertheless, a comprehensive analysis of how the concept of virtual particles was received in the Soviet Union, and its potential implications for Marxist philosophy of science, merit more detailed study that are beyond the scope of this paper. Novozhilov 1959.

n49 Falkenburg 2007, on 236.

n50 Hesse 1961, on 277.

n51 Ibid.

n52 Ibid., on 278.

n53 Ford 1963, on 168.

n54 Ibid., on 171.

n55 Ibid., on 207 and 209.

n56 The letter was rejected by *Science*. It has, however, been reproduced in LeShan and Margenau 1982, on 256–257.

n57 Margenau 1954a, on 82; Margenau 1954b.

n58 Henry Margenau, “Article on Fields for *Encyclopædia Britannica*,” undated, Henry Margenau Papers (MS 129), Manuscripts and Archives, Yale University Library, box 2, folder 35 (“Fields, undated”). The entry appeared in volume 7 of the 1974 edition of the encyclopedia. It was, however, amputated of its section on relativistic quantum fields. Margenau 1974.

n59 This situation is described in more detail in: Gross 2004; Borrelli 2018.

n60 Fermi 1934.

n61 The early realization of the limitations of the techniques developed in the late 1940s is notoriously illustrated by a letter from Feynman to Enrico Fermi, dated 19 December 1951, containing the following endnote: “Don't believe any calculations in meson theory which uses a Feynman diagram!” Enrico Fermi Collection, Hanna Holborn Gray Special Collections Research Center, University of Chicago Library, box 9, folder 25 (“Feynman, R. P.”).

n62 On the development of the S‐Matrix theory, see: Cushing 1990; Blum 2017.

n63 Gell‐Mann 1964; Zweig 1964a and b.

n64 On this episode, see: Kaiser 2011; Freire Jr. 2014.

n65 Henry Margenau, “Article on Fields for *Encyclopædia Britannica*,” undated, Henry Margenau Papers (MS 129), Manuscripts and Archives, Yale University Library, box 2, folder 35 (“Fields, undated”).

n66 Bunge 1970, on 507.

n67 Ibid., on 508.

n68 Ibid.

n69 More in: Gross 2004; Borrelli 2018.

n70 Kuhn 1962.

n71 Shrader‐Frechette 1977.

n72 Ibid., on 409.

n73 Hendrick and Murphy 1981; Weingard 1982.

n74 A notable exception is Arthur 2012.

n75 Ehberger 2024b. From a historiographical point of view, one might also be inclined to consider virtual particles as “epistemic things” or “epistemic objects.” Despite the challenges posed by their unclear ontological and experimental status, this perspective would underscore their role as fundamental objects of research, especially considering their evolving trajectory as mind representations. Interested readers are encouraged to explore Urko Gorriñobeaskoa Artolozaga's contribution to the present issue of *Berichte zur Wissenschaftsgeschichte* for further insights.

n76 Ibid. Note that this incentive is in no way intended to be normative.

## References

[1] Aitchson, Ian J. R., and Anthony J. G. Hey, *Gauge Theories in Particle Physics: A Practical Introduction. Volume 1: From Relativistic Quantum Mechanics to QED*, 4th edn. (Boca Raton: CRC Press, 2013).

[2] Anderson, Carl D. , and Seth Neddermeyer , “Cloud Chamber Observations of Cosmic Rays at 4300 Meters Elevation and Near Sea-Level,” Physical Review 50, no. 4 (1936): 263–271.

[3] Arthur, Richard T. , “Virtual Processes and Quantum Tunnelling as Fictions,” Science & Education 21 (2012): 1461–1473.

[4] Beck, Guido , “Über die Streuung von Teilchen durch Kraftfelder,” Zeitschrift für Physik 62 (1930): 331–351.

[5] Bhabha, Homi J. , “Nuclear Forces, Heavy Electrons and the β-Decay,” Nature 141, no. 3559 (1938): 117–118.

[6] Blum, Alexander S. , “The State Is Not Abolished, It Withers Away: How Quantum Field Theory Became a Theory of Scattering,” Studies in History and Philosophy of Science Part B: Studies in History and Philosophy of Modern Physics 60 (2017): 46–80.

[7] Blum, Alexander S. , and Martin Jähnert , “Real Virtuality and Actual Transitions: Historical Reflections on Virtual Entities Before Quantum Field Theory,” Perspectives on Science 32, no. 3 (2024): 329–349.

[8] Borrelli, Arianna , “The Weinberg-Salam Model of Electroweak Interactions: Ingenious Discovery or Lucky Hunch?” Annalen der Physik 530, no. 2 (2018): 1700454.

[9] Brown, Laurie M. , “Yukawa's Prediction of the Meson,” Centaurus 25 (1981): 71–132.

[10] Bunge, Mario , “Virtual Processes and Virtual Particles: Real or Fictitious?” International Journal of Theoretical Physics 3, no. 6 (1970): 507–508.

[11] Cassidy, David C. , “Cosmic Ray Showers, High Energy Physics, and Quantum Field Theories: Programmatic Interactions in the 1930s,” Historical Studies in the Physical Sciences 12, no. 1 (1981): 1–39.

[12] Cushing, James T., *Theory Construction and Selection in Modern Physics: The S Matrix* (Cambridge: Cambridge University Press, 1990).

[13] Dirac, Paul A. M. , “The Quantum Theory of the Emission and Absorption of Radiation,” Proceedings of the Royal Society of London A: Mathematical, Physical, and Engineering Sciences 114, no. 767 (1927a): 243–265.

[14] Dirac, Paul A. M. , “The Quantum Theory of Dispersion,” Proceedings of the Royal Society of London A: Mathematical, Physical, and Engineering Sciences 114, no. 769 (1927b): 710–728.

[15] Ehberger, Markus, “I'm Not There. Or: Was the Virtual Particle Ever Born?” in *Biographies in the History of Physics: Actors, Objects, Institutions*, ed. Christian Forstner and Mark Walker (Cham: Springer, 2020), 261–280.

[16] Ehberger, Markus , “‘The Language of Dirac's Theory of Radiation’: The Inception and Initial Reception of a Tool for the Quantum Field Theorist,” Archive for History of Exact Sciences 76 (2022): 531–571.

[17] Ehberger, Markus, “From Virtual Oscillators to Virtual Transitions to Virtual Particles: Practices and Representations in the Formation of the Virtual Particle Concept” (PhD thesis, TU Berlin, 2024a).

[18] Ehberger, Markus , “How to Study Virtual Entities Historically? A Proposal,” Perspectives on Science 32, no. 3 (2024b): 278–299.

[19] Euler, Hans, and Bernhard Kockel, “Über die Streuung von Licht an Licht nach der Diracschen Theorie,” *Die Naturwissenschaften* 23, no. 15 (1935): 246–247.

[20] Euler, Hans , “Über die Streuung von Licht und Licht nach der Diracschen Theorie,” Annalen der Physik 418, no. 5 (1936): 398–448.

[21] Falkenburg, Brigitte, *Particle Metaphysics: A Critical Account of Subatomic Reality* (Berlin: Springer, 2007).

[22] Fermi, Enrico , “Versuch einer Theorie der β-Strahlen. I,” Zeitschrift für Physik 88, no. 3 (1934): 161–177.

[23] Feynman, Richard , “Space-Time Approach to Quantum Electrodynamics,” Physical Review 76, no. 6 (1946): 769–789.

[24] Ford, Kenneth W., *The World of Elementary Particles* (New York: Blaisdell, 1963).

[25] Fox, Tobias , “Haunted by the Spectre of Virtual Particles: A Philosophical Reconsideration,” Journal for General Philosophy of Science 39, no. 1 (2008): 35–51.

[26] Freire Jr., Olival, *The Quantum Dissidents: Rebuilding the Foundations of Quantum Mechanics (1950–1990)* (Berlin: Springer, 2014).

[27] Frenkel, Yakov I. , “The Quantum Theory of the Absorption of Light,” Nature 124 (1929a): 758–759.

[28] Frenkel, Yakov I. , “Über quantenmechanische Energieübertragung zwischen atomaren Systemen,” Zeitschrift für Physik 58, no. 11 (1929b): 794–804.

[29] Frenkel, Yakov I., *Wave Mechanics: Advanced General Theory* (Oxford: Clarendon Press, 1934).

[30] Gamow, Georges, and Charles L. Critchfield, *Theory of Atomic Nucleus and Nuclear Energy-Sources* (Oxford: Clarendon Press, 1949).

[31] Gell-Mann, Murray , “A Schematic Model of Baryons and Mesons,” Physics Letters 8, no. 3 (1964): 214–215.

[32] Gross, David J. , “Asymptotic Freedom and QCD–a Historical Perspective,” Nuclear Physics B-Proceedings Supplements 135 (2004): 193–211.

[33] Halzen, Francis, and Alan D. Martin, *Quarks and Leptons: An Introductory Course in Modern Particle Physics* (New York: Wiley and Sons, 1984).

[34] Harlander, Robert V., Sven Y. Klein, and Maximilian Lipp, “FeynGame,” *Computer Physics Communications* 256 (2020): 107465.

[35] Harlander, Robert V. , and Jean-Philippe Martinez , “The Development of Computational Methods for Feynman Diagrams,” The European Physical Journal H 49, no. 1 (2024): 4.

[36] Hesse, Mary B., *Forces and Fields* (London: T. Nelson, 1961).

[37] Heitler, Walter , “II. Cosmic Rays,” Reports on Progress in Physics 5 (1938): 361–389.

[38] Heisenberg, Werner, “Bemerkungen zur Theorie des Atomkerns [1935],” in *Scientific Review Papers, Talks, and Books / Wissenschaftliche Übersichtsartikel, Vorträge und Bücher*, Gesammelte Werke / Collected Works, vol. B, ed. Walter Blum, Hans-Peter Dürr, and Helmut Rechenberg (Berlin and Heidelberg: Springer, 1984), 238–246.

[39] Heisenberg, Werner , “Zur Theorie der explosionsartigen Schauer in der kosmischen Strahlung. II,” Zeitschrift für Physik 113 (1939): 61–86.

[40] Hendrick, R. Edward, and Anthony Murphy, “Atomism and the Illusion of Crisis: The Danger of Applying Kuhnian Categories to Current Particle Physics,” *Philosophy of Science* 48, no. 3 (1981): 454–468.

[41] Jaeger, Gregg , “Are Virtual Particles Less Real?” Entropy 21 (2019): 141. 33266857 10.3390/e21020141 PMC7514619

[42] Kaiser, David, *Drawing Theories Apart: The Dispersion of Feynman Diagrams in Postwar Physics* (Chicago: University of Chicago Press, 2005).

[43] Kaiser, David, *How the Hippies Saved Physics: Science, Counterculture, and the Quantum Revival* (New York: WW Norton & Company, 2011).

[44] Kojevnikov, Alexei , “Freedom, Collectivism, and Quasiparticles: Social Metaphors in Quantum Physics,” Historical Studies in the Physical and Biological Sciences 29, no. 2 (1999): 295–331.

[45] Kramers, Hendrik A. , and Werner Heisenberg , “Über die Streuung von Strahlung durch Atome,” Zeitschrift für Physik 31, no. 1 (1925): 681–708.

[46] Kuhn, Thomas S., *The Structure of Scientific Revolutions* (Chicago: University of Chicago Press, 1962).

[47] Landau, Lev , and Rudolf Peierls , “Erweiterung des Unbestimmtheitsprinzips für die relativistische Quantentheorie,” Zeitschrift für Physik 69, no. 1 (1931): 56–69.

[48] Lattes, Cesare M. G., Hugh Muirhead, Giuseppe P. S. Occhialini, and Cecil F. Powell, “Processes Involving Charged Mesons,” *Nature* 159 (1947): 694–697.

[49] LeShan, Lawrence, and Henry Margenau, *Einstein's Space and Van Gogh's Sky: Physical Reality and Beyond* (New York: MacMillan Publishing Company, 1982).

[50] Margenau, Henry , “Causality in Quantum Electrodynamics,” Diogenes 2, no. 6 (1954a): 74–84.

[51] Margenau, Henry , “Can Time Flow Backwards?” Philosophy of Science 21, no. 2 (1954b): 79–92.

[52] Margenau, Henry, “Fields, Theory of, in Physics,” in *The New Encyclopædia Britannica*, vol. 7 (Chicago: William Bentonm Publisher, 1974), 293–295.

[53] Marshak, Robert E., and Hans A. Bethe, “On the Two-Meson Hypothesis,” *Physical Review* 72, no. 6 (1947): 506–509.

[54] Martinez, Jean-Philippe , “Virtuality in Modern Physics in the 1920s and 1930s: Meanings of an Emerging Notion,” Perspectives on Science 32, no. 3 (2024): 350–371.

[55] Monaldi, Daniela , “Life of μ: The Observation of the Spontaneous Decay of Mesotrons and Its Consequences,” Annals of Science 62, no. 4 (2005): 419–455.

[56] Novozhilov, Yuri V., *Elementarnye Chastitsy* (Moscow: Gosudarstvennoye izdatelstvo fiziko-matematicheskoy literatury, 1959).

[57] Peierls, Rudolf E., Abdus Salam, Paul T. Matthews, and Gordon Feldman, “A Survey of Field Theory,” *Reports on Progress in Physics* 18, no. 1 (1955): 423.

[58] Raman, Chandrashekhara V. , “Investigation of Molecular Structure by Light Scattering,” Transactions of the Faraday Society 25 (1929): 781–792.

[59] Rorvig, Morchedai, “OK, WTF Are ‘Virtual Particles’ and Do They Actually Exist?” *Vice*, 11 May 2020, online: https://www.vice.com/en/article/3az8 g3/ok-wtf-are-virtual-particles-and-do-they-actually-exist (accessed 6 February 2024).

[60] Rosenfeld, Léon, *Nuclear Forces*, vol. 1 (Amsterdam: North-Holland Publishing Company, 1948).

[61] Rueger, Alexander , “Attitudes Towards Infinities: Responses to Anomalies in Quantum Electrodynamics, 1927–1947,” Historical Studies in the Physical and Biological Sciences 22, no. 2 (1992): 309–337.

[62] Schweber, Silvan S., *QED and the Men Who Made It: Dyson, Feynman, Schwinger and Tomonaga* (Princeton: Princeton University Press, 1994).

[63] Shrader-Frechette, Kristin , “Atomism in Crisis: An Analysis of the Current High Energy Paradigm,” Philosophy of Science 44, no. 3 (1977): 409–440.

[64] Valente, Mario B. , “Are Virtual Quanta Nothing but Formal Tools?” International Studies in the Philosophy of Science 25, no. 1 (2011): 39–53.

[65] Weingard, Robert , “Do Virtual Particles Exist?” PSA: Proceedings of the Biennial Meeting of the Philosophy of Science Association 1982, no. 1 (1982): 235–242.

[66] Wick, Giancarlo C. , “Range of Nuclear Forces in Yukawa's Theory,” Nature 142 (1938): 993–994.

[67] Yukawa, Hideki , “On the Interaction of Elementary Particles. I,” Proceedings of the Physico-Mathematical Society of Japan 17 (1935): 48–57.

[68] Yukawa, Hideki , and Shoichi Sakata , “On the Interaction of Elementary Particles. II,” Proceedings of the Physico-Mathematical Society of Japan 19 (1937): 1084–1093.

[69] Zweig, George, *An SU_3_ Model for Strong Interaction Symmetry and Its Breaking*, report no. CERN-8182/TH-412 (1964a).

[70] Zweig, George, *An SU_3_ model for strong interaction symmetry and its breaking II*, report no. CERN-8419/TH-412 (1964b).

